# Carbon monoxide dehydrogenase-encoding microorganisms in volcanic astrobiological analogues: an enzyme system to investigate the evolution of life

**DOI:** 10.1093/femsec/fiag022

**Published:** 2026-02-25

**Authors:** Vito Latorre, Xabier Vázquez-Campos, Belinda Ferrari, Marcela Hernández

**Affiliations:** School of Biological Sciences, University of East Anglia, Norwich, NR4 7TJ, United Kingdom; School of Biotechnology and Biomolecular Sciences, University of New South Wales, Sydney 2052, Australia; School of Biotechnology and Biomolecular Sciences, University of New South Wales, Sydney 2052, Australia; School of Biological Sciences, University of East Anglia, Norwich, NR4 7TJ, United Kingdom

**Keywords:** carbon monoxide, CO dehydrogenase, soil microbes, volcanic deposits, exoplanets, maintenance energy

## Abstract

Volcanic environments provide analogues for studying the origin of life and its persistence under extreme conditions on early Earth and other planetary bodies. Pioneering microbes that oxidise inorganic gases, such as carbon monoxide (CO), provide energy for survival and initiate primary succession. Similar geological and atmospheric conditions shaped by volcanism, meteoritic impacts, and tidal heating have existed, or still exist, on Mars, Venus, and icy moons, where CO may serve as a metabolic substrate. This review explores the evolutionary significance of CO dehydrogenase (CODH), an enzyme responsible for the oxidation of CO to carbon dioxide, thereby linking geochemical energy fluxes to the emergence of biological carbon. Genomic evidence from eight globally distributed volcanic sites confirms the presence of genes encoding CODH. Genes encoding aerobic CO oxidation (*coxL*) were consistently abundant and conserved, whereas genes encoding anaerobic oxidation (*cdh*- and *coo*-related genes) showed site-specific dominance and variability, reflecting differences in microbial community composition and environmental conditions. At Poás Volcano, several taxa, particularly members of Desulfobacterota, exhibited genetic versatility across nine gene clusters, highlighting their adaptive capacity. These findings demonstrate how trace gas metabolism can support microbial survival in volcanic soils, providing insight into potential habitability on other planetary bodies.

## Teaser

Volcanic ecosystems reveal how ancient CO-oxidizing microbes evolved CODH genes, enabling energy generation and adaptive versatility across diverse extreme environments on Earth and other planetary bodies influenced by geochemical processes.

## Introduction

In the last decade, the search for life beyond Earth has increasingly focused on environments that are geochemically active and extremely poor in organic carbon. Understanding microbial survival and metabolism in such systems broadens our concept of habitability beyond the traditional framework, which holds that the building blocks of life are liquid water and organic compounds (Cockell et al. 2016). Instead, these environments, characterised by a combination of redox gradients and high energy availability, enable reactions between otherwise inert compounds such as carbon monoxide (CO), sulfur dioxide (SO_2_), and hydrogen sulfide (H_2_S), which can serve as alternative energy sources for life (Cordero et al. 2019, Greening & Grinter, 2022, Dede et al. 2024). Such chemolithotrophic metabolism, including CO and sulfur oxidation, have been documented in terrestrial analogue habitats, emphasising their ecological importance under extreme conditions (King 2007, Ray et al. 2022, Magnuson et al. 2023, Dede et al. 2024). Recent planetary missions, such as NASA’s Perseverance rover exploring Martian sediments, and the proposed detection of potential biosignatures in the Venusian atmosphere, have strengthened the relevance of these geochemically dynamic settings as natural laboratories for testing the limits of life (Greaves et al. 2021, Hart and Cardace 2023). Studying such systems provides critical insights into how metabolism and ecological networks might evolve on other worlds, including Mars, Venus, and the icy moons of the outer solar system.

Volcanic environments on Earth offer critical analogues for studying these processes in an accessible real-world context. Fresh lava flows, tephra deposits, and pyroclastic materials create sterile surfaces that are initially devoid of organic carbon and are exposed to thermal fluctuations, desiccation, and toxic volcanic emissions (Schmidt et al. 2018). Despite these harsh conditions, microbial life rapidly colonises volcanic soils, initiating ecological succession and shaping early biogeochemical cycles (Hernández et al. 2020). Early colonisers often rely on chemolithoautotrophic metabolism, oxidising reduced gases such as CO, hydrogen (H_2_), and H_2_S to obtain energy in the absence of sunlight or organic nutrients (King 2007). Over time, these pioneering microbes weather mineral surfaces, promote the formation of microhabitats, and contribute to the gradual establishment of more complex microbial consortia (Fantom et al. 2025). Understanding these initial colonisation processes provides valuable clues to how life might take hold on freshly formed planetary crusts or in subsurface volcanic regions of other planets.

The oxidation of CO in microorganisms is facilitated by CO dehydrogenase (CODH), a highly conserved enzyme that catalyses the reversible transformation of CO to carbon dioxide (CO_2_), allowing microbes to utilise CO as an energy source. Genes encoding CODH are widespread among microorganisms inhabiting volcanic and other geochemically active environments, where organic carbon is scarce and trace gases constitute a significant energy source. Due to its long evolutionary history and central role in trace gas metabolism, CODH represents an effective functional marker for investigating microbial survival strategies in volcanic soils and their relevance as astrobiological analogues.

In this review, we analysed metagenomic data from freshly deposited soils of volcanic origin across eight globally distributed sites, spanning diverse climatic and geological conditions including Llaima Volcano and Atacama Desert (Chile), Poás Volcano (Costa Rica), Mt. Melbourne (Antarctica), Pantelleria Volcano (Italy), Golden Dome Cave (California), Mauna Loa Volcano (Hawaii), and Svalbard (Norway). To obtain a broad comparative perspective, we compiled and reanalysed publicly available metagenome-assembled genomes (MAGs) that were retrieved from environments with a range of temperatures, altitudes, and mineralogical compositions. The aim of our review is to explore the biochemical, ecological, and evolutionary significance of potential CODH-utilising microbes in volcanic environments, to evaluate geothermically active terrestrial sites as analogues, and to discuss their implications for astrobiology and the search for life beyond Earth. By identifying key metabolic pathways, such as those linked to CO oxidation, this review also seeks to refine potential biosignature targets and inform life-detection strategies for future planetary exploration missions.

## Astrobiological context

Volcanism has significantly modified the surfaces of terrestrial planets in the inner solar system. Other processes, such as soil dynamics and gravitational collapse, cause fracturing and faulting even on smaller bodies like asteroids and natural satellites, but volcanism and tectonism have had relatively minor effects on these smaller bodies (Platz et al. 2015). Thus, the presence of these phenomena, even if limited in magnitude, is widespread across diverse planetary objects.

The role of volcanic environments in shaping planetary surfaces, outgassing volatiles that drive atmospheric composition, and creating dynamic chemical gradients makes them critical targets in the search for extraterrestrial life (Zilinskas et al. 2022, Maurice et al. 2024). Venus, the second planet from the Sun, has a surface temperature as high as 464 °C (Lebonnois and Schubert 2017). Although currently inhospitable, in its early history, the planet may have sustained liquid water, providing a primary substrate capable of supporting life (Cockell et al. 2016, Westall et al. 2023). Thermochemical models have demonstrated that prolonged silicate weathering on relatively stable basalts could have buffered atmospheric CO₂, enabling the decarbonation of the crust (Höning et al. 2021). Such atmospheric and crustal dynamics might have created transient environments that favoured the accumulation of CO, a potential energy source for early microbial life forms harbouring CODH enzymes or similar metabolic pathways.

Recent orbital and rover observations have recorded preserved evidence of past volcanism in Jezero Crater on Mars. These observations identified a conical edifice on Jezero’s southeastern rim whose morphometry, thermal inertia, crater retention, and mineralogical signatures are consistent with a composite volcano, potentially contributing to habitable conditions in the planet’s past (Cuevas-Quiñones et al. 2025, Hurowitz et al. 2025). Previous Mars missions have broadly described atmospheric and sedimentary processes (Mahaffy et al. 2013, Grotzinger 2014); however, Jezero provides a specific environment where volcanically influenced substrates can be clearly associated with previous habitability (Wiens et al. 2022). Martian surface and igneous rocks appear to be mostly tholeiitic basalts reflecting extensive mantle-derived volcanism, with little evidence for silica-rich or highly evolved magmatic compositions (McSween Jr et al. 2009, Hurowitz et al. 2025). Data from Curiosity rover’s mission have indicated that the Martian atmosphere is composed of five major atmospheric components in specific mixing ratios (CO_2_, Ar, N_2_, O_2_, and CO) (Mahaffy et al. 2013). CO is a minor species in the present Martian gas mixture, produced at high altitude (80-100 km) by photolysis of the main atmospheric CO_2_ constituent (Tarnas et al. 2018, Ashimananda et al. 2025). Subsurface or vent-proximal environments may offer buffered niches where CO-oxidising microorganisms could persist despite the thin, CO_2_-dominated atmosphere, low atmospheric pressure, and chemically demanding regolith conditions that characterise the modern Martian surface (King 2015, Myers and King 2017, Wordsworth et al. 2021). This is consistent with experimental evidence showing that terrestrial microbes can oxidise CO under low-pressure, saline, and suboxic conditions relevant to the Mars regolith (King 2015). Laboratory incubations have demonstrated that the halophilic Gammaproteobacteria *Alkalilimnicola ehrlichii* MLHE-1 maintains CO oxidation even under suboxic and CO_2_-rich atmospheres, simulating Martian conditions. This reinforces the idea that trace metabolism could persist in near-surface or vent-proximal niches on Mars (King 2015). Outside the Solar System, exoplanets with active volcanism or thin, gas-rich atmospheres may similarly host environments where microbes capable of oxidising trace gases could dominate (Misra et al. 2015, Cowan et al. 2022).

The icy moons Europa and Enceladus, which orbit the gas giants Jupiter and Saturn, respectively, are characterised by extreme conditions associated with geothermal activity. Analyses of plume material revealed the presence of CO, CO_2_, and H_2_, which could serve as energy sources for microbial life capable of trace-gas metabolism within subsurface oceans (Matson et al. 2007). Analogues, such as Mount Melbourne in Antarctica — a basaltic and trachyandesitic, ice-covered stratovolcano — present a rare confluence of volcanic heat and polar cold that could sustain habitable niches. Such an analogue could aid the preservation of potential biosignatures (Myeong et al. 2024). Indeed, metagenomic studies have shown that these soils host unique microbial communities capable of thermophilic and chemolithotrophic specialised metabolic pathways, such as multiple autotrophic carbon fixation routes (e.g. CBB cycle, rTCA cycle) and sulfur oxidation (e.g. thiosulfate) (Park et al. 2021).

## Early Earth as a volcanic world

Volcanism on both Mars and Earth, from approximately 4.3 billion years (Ga) ago to around 3.1 Ga for Mars, played a critical role in shaping the chemical landscape. Therefore, volcanism potentially provided the essential gases and energy fluxes required to fuel the complex chemical reactions underpinning prebiotic pathways (Sasselov et al. 2020). The emergence of life on Earth is fundamentally linked to the planet’s early geological and atmospheric evolution, which created the necessary conditions for these chemical processes to give rise to living systems as we know them.

During Earth’s early Hadean eon, extensive volcanic activity and global magma ocean phases likely emitted CO, H_2_, methane (CH_4_), and CO_2_ into a transient atmosphere, while organic carbon was scarce, conditions that may have set the stage for prebiotic chemistry (Lichtenberg et al. 2021). The redox state of the mantle played a critical role in determining the relative abundance of outgassed species, particularly CO and H_2_, as illustrated in recent modelling of volcanic gas fluxes under varying mantle oxidation conditions (Guimond et al. 2021). The magnitude of this redox control has been demonstrated, with CO more abundant under strongly reduced conditions, and cumulative outgassed masses exceeding those of CO_2_, underscoring the potential for CO-rich early atmospheres (Guimond et al. 2021).

In laboratory experiments, volcanic ash and meteoritic particles have been shown to catalyse the conversion of CO_2_ into organic precursors (alcohols, aldehydes, hydrocarbons) under early Earth–like conditions (Peters et al. 2023). This suggests that volcanic material was not merely a passive landscape but actively promoted chemical reactions that may have contributed to the emergence of life (Peters et al. 2023). This catalytic activity not only contributed to the synthesis of organic molecules but also the generation of reduced gases such as CO, which could have later served as an energy substrate for early anaerobic carbon-fixing microorganisms. By examining fresh volcanic deposits, researchers are gaining insight into the evolution of microbial metabolism and the conditions under which life could arise to infer ecological strategies that early bacteria may have employed to thrive in low-carbon, high-gas environments.

## Volcanic analogues on Earth

The relevance of volcanic environments on Earth stems not only from their dynamic geological features, such as morphology and composition, which have been confirmed as plausible planetary analogues, but also from their ability to host microbial life that thrives under extreme conditions. The Central Cordillera in Costa Rica, is a massive orogenic system that includes the basaltic-andesite stratovolcano of Poás Volcano. This structure exhibits active fumaroles and hydrothermal alteration in basaltic-andesitic substrate, producing minerals such as natroalunite, jarosite, gypsum, and hydrated silica (including the disordered mixture cristobalite-tridymite known as opal-CT), which are also found in Martian alteration sites (Hynek et al. 2018). In particular, Laguna Caliente crater lake, with its extremely acidic pH (−1 to ∼1.5), high sulfur emissions from frequent phreatic eruptions, and temperature fluctuations (19 °C to ∼90 °C), hosts extremely low microbial diversity, dominated by acidophilic chemolithotrophs (Hynek et al. 2018).

Fumarolic soils are also present in the high-elevation volcanic debris fields of the Atacama Desert in Chile, where dry oxidised basaltic tephra is exposed to high UV flux, low atmospheric shielding, and strong temperature fluctuations (Schmidt et al. 2018). This makes the site one of the most Mars-like locations on Earth, especially when compared with surface conditions inferred for early Mars during periods of enhanced volcanism and limited atmospheric shielding (Schmidt et al. 2018). At high altitude, fumaroles provide more stable, warmer temperatures and more reliable water supplies, supporting trophically complex microbial communities comparable to the diverse soil communities found at lower elevations. This includes the proliferation of extremophiles, in particular eukaryotes such as *Naganishia* spp. that can withstand UV radiation, cold, and large temperature fluctuations (Schmidt et al. 2018).

An additional geothermal-polar analogue that has revealed trace gas microbial strategies is Mount Erebus in the Antarctic region. This volcanic system has a stable, convecting phonolitic lava lake characterised by continuous surface overturn and thermal emissions exceeding ∼827 °C. The lake displays morphological and radiative features similar to those found at the Pele lava lake on Jovian moon Io (Davies et al. 2008). Furthermore, the formation of ice towers and fumarolic chimneys in this polar volcanic area, through the sublimation and deposition of volcanic gases (particularly H_2_O, CO_2_, and SO_2_), establishes thermally buffered microhabitats with internal temperatures remaining above freezing, despite external conditions dropping below –50 °C. These environments parallel hypothesised subglacial and fumarolic refugia on early Mars (Hoffman and Kyle 2003). Under such physicochemical conditions, a putative CODH gene cluster (*coxMLS*) has been identified within the genome of *Candidatus* Aramenus erebusense, a facultatively anaerobic heterotroph capable of surviving in cold, oligotrophic, and redox-variable conditions (Herbold et al. 2024). The presence of CODH genes suggests a potential capacity for trace-gas metabolism, specifically CO oxidation, which may contribute to energy acquisition in ecosystems with limited organic substrates (Tebo et al. 2015).

## Microbial colonisation of fresh volcanic soils

The freshly deposited substrates produced during volcanic eruptions are initially sterile, characterised by unstable physical structures and low organic carbon content. Pioneering microorganisms, capable of using inorganic energy sources, drive primary succession under these conditions (Fantom et al. 2025). In addition to ensuring their own survival, these pioneers facilitate the gradual transformation of the substrate into soil capable of supporting more complex ecosystems.

Among the predominant bacterial phyla found in early volcanic soils are Actinomycetota, Pseudomonadota, and Chloroflexota (Fantom et al. 2025). Actinomycetota, and especially members of class Actinomycetia, such as *Mycolicibacterium smegmatis* (formerly *Mycobacterium smegmatis*), are renowned for their tolerance to anaerobic or hypoxic stress via fermentative hydrogen production (Berney et al. 2014). In contrast, some Pseudomonadota members display strong adaptations to low–oxygen or fluctuating redox conditions, partly via changes in the quinone pathway (Chobert et al. 2025). These species frequently possess genes encoding CODH, which facilitate high-affinity CO oxidation and provide energy for cell maintenance in nutrient-limited conditions (Hernández et al. 2020). These microbial communities can efficiently absorb ambient CO in pristine soils, as demonstrated by experimental analysis of Hawaiian volcanic deposits, where measurable uptake rates have been observed within days of deposition, ranging approximately 0.2 to 5 mg CO m ^−2^ day ^−1^ (King and Weber 2008).

CO oxidisers have an ecological function that goes beyond obtaining energy. These microorganisms contribute to the carbon input in barren, carbon-limited environments by oxidising CO to CO_2_, or in the Calvin–Benson–Bassham (CBB) cycle for the CO_2_ fixation. Thus, CO-oxidisers are likely to support the growth of early autotrophs and heterotrophic microbes (Tebo et al. 2015, Hernández et al. 2020). Over time, this results in the formation of more functionally diverse, structured microbial communities, preparing the way for vascular plants, lichens, and mosses to colonise.

## CODH: biochemical and genetic foundations

CODH is a highly conserved metalloenzyme that catalyses a reversible redox reaction in which CO is oxidised using water as the electron donor to form CO_2_, releasing two protons and two electrons. This fundamental reaction is relevant to both biological systems (e.g. CO oxidation by enzymes like CODH) and abiotic chemical processes, including electrochemical experiments on redox catalysis (Wang et al. 2013). This enzymatic reaction serves as a central component in the microbial carbon cycle, mediating a significant ecological and evolutionary role in oxidation and reduction processes under both aerobic and anaerobic conditions (Bährle et al. 2023). Two principal types of CODH enzymes have been identified based on their metal cofactors and oxygen sensitivity: the oxygen-sensitive nickel-CODH (Ni-CODH) typically found in anaerobic bacteria and archaea, either as an independent enzyme or as part of a larger complex with acetyl-coenzyme A (CoA-SH) synthase/decarbonylase (ACS), and the oxygen-tolerant CODHs containing the molybdopterin/Cu/Fe–S cluster (MoCu-CODH), predominantly present in aerobic prokaryotes (Boer et al. 2014).

Functionally, CODH plays dual roles in microbial metabolism. In anaerobic carboxydotrophs, CODH is linked to the carbon fixation pathways of the Wood–Ljungdahl pathway or the CBB cycle. These microorganisms can utilise CO as their sole carbon source and as a reductant to fix the resulting CO_2_. The CO produced by CODH serves as a substrate for acetyl-CoA synthase (ACS), which combines it with a methyl group and coenzyme A to form acetyl-CoA, thereby linking carbon fixation directly to energy conservation and biosynthesis. In this context, CODH mediates both the generation of reducing equivalents and the transfer of carbon, allowing these organisms to utilise CO_2_ as their sole carbon source while producing ATP and reducing equivalents NAD(P)H + H^+^. In contrast, in carboxydovores, CODH is primarily used for energy acquisition, feeding electrons into respiratory chains or other electron acceptors such as O_2_, nitrate, or sulfate. In some cases, CODH also supports the CBB cycle by supplying CO_2_ via low-affinity CODH enzymes, but relying on other carbon sources for biosynthesis (King and Weber 2007, Cordero et al. 2019). In these cases, CODH provides a source of energy that allows microorganisms to survive in harsh conditions over extended periods.

The genetic architecture of CODHs reflects their deep evolutionary divergence and functional specialisation. Aerobic MoCu-CODHs are encoded by *cox* genes (Cunliffe 2011), in which *coxL* encodes the large catalytic subunit, *coxM* the medium subunit, and *coxS* the small electron-transfer subunit. Two major forms of *coxL* have been described: form I represents the canonical catalytic enzyme responsible for CO oxidation under oxic conditions using quinones as electron acceptors (Bährle et al. 2023, Fantom et al. 2025), while form II is more divergent, often acting in regulatory or accessory roles rather than direct CO oxidation (Bährle et al. 2023).

In contrast, [NiFe]-CODHs, central to CO_2_ reduction and CO formation, are encoded by *acs, cdh*, and *coo* operons depending on their functional context (Dent et al. 2023). The *coo* system encodes CODH along with accessory proteins needed to liberate energy from CO oxidation when coupled with proton reduction to form H_2_ or another reductive process. Generally, it includes *cooS*, encoding the catalytic subunit containing the Ni–Fe–S active site, *cooF*, encoding an iron–sulfur electron carrier, and *cooC*, a nickel-insertion chaperone required for enzyme maturation (Dent et al. 2023). In many methanogens and acetogens, the CODH/ACS operon consists of a five subunits Ni–Fe–S enzyme complex of the Wood–Ljungdahl pathway. *cdhA*/*acsA* catalyses CO₂ reduction to CO, *cdhC*/*acsB* forms acetyl-CoA from CO and a methyl group, and *cdhD*–*cdhE*/*acsD*-*acsE* provide the methyl via a corrinoid iron–sulfur complex (Adam et al. 2018).

**Figure 1:**
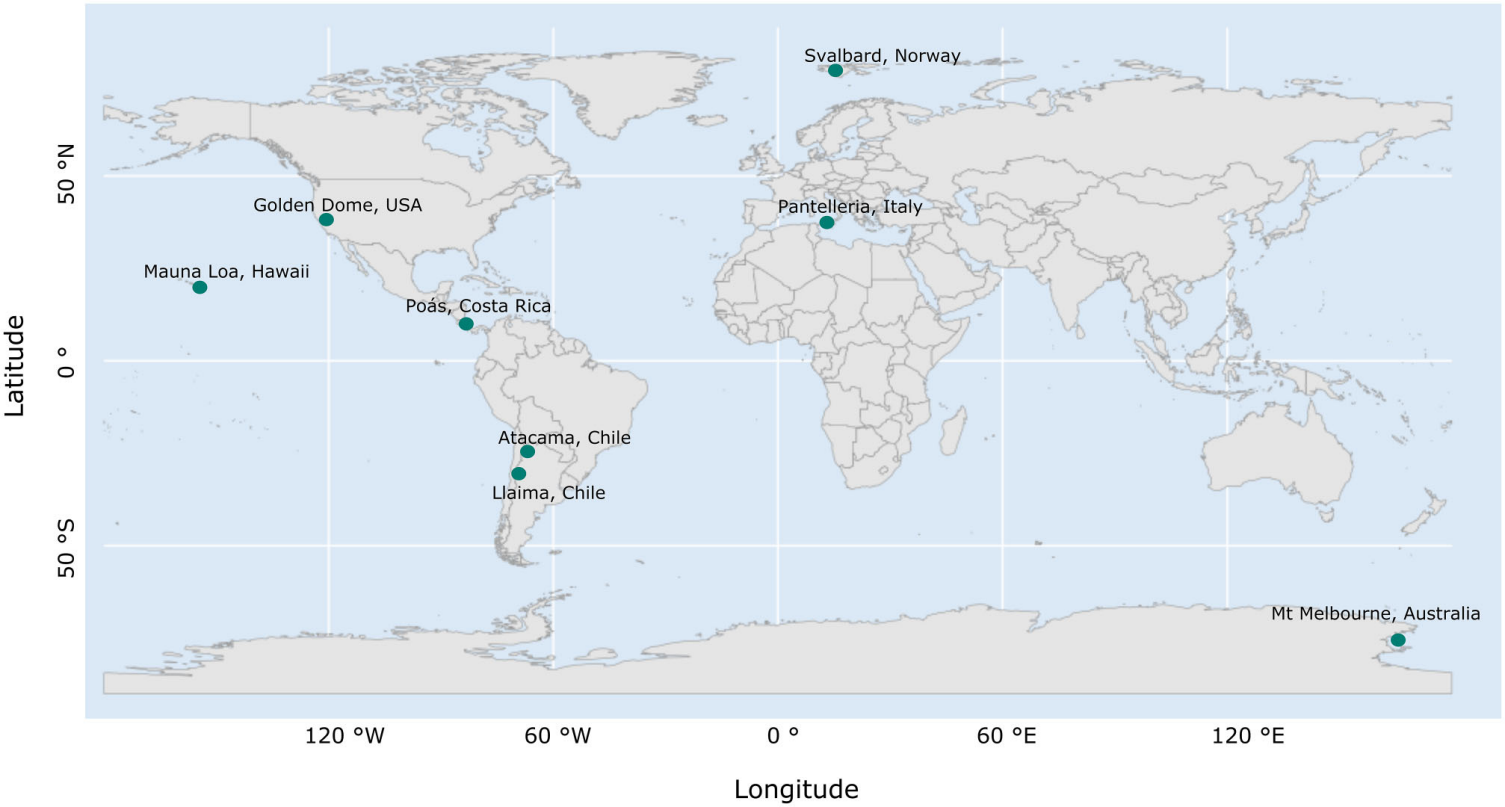
Global map displaying the geographic locations of selected volcanic sites relevant to our study. The sites span a wide range of latitudes and longitudes across the globe. Included locations are: Llaima Volcano and Atacama Desert (Chile), Poás Volcano (Costa Rica), Mount Melbourne (Antarctica), Pantelleria Volcano (Italy), Golden Dome Cave (California, USA), Mauna Loa Volcano (Hawaii, USA), and Svalbard (Norway).

This metabolic versatility highlights the potential for life to persist in environments characterised by geochemical disequilibria and minimal carbon input, supporting the astrobiological relevance of volcanic terrains in the search for biosignatures. We conducted a thorough systematic review of the literature and examined publicly accessible metagenomic datasets from volcanic-origin soils around the world. Our goal was to investigate the global distribution and functionality of CODH enzymes in volcanic astrobiological analogues. Eight volcanic origin sites, considered key astrobiological analogues, were selected for our analysis: Llaima Volcano (Hernández et al. 2020) and Atacama Desert (Chile) (Andreani-Gerard et al. 2024), Poás Volcano (Costa Rica) (Rogers et al. 2023), Mount Melbourne (Antarctica) (Myeong et al. 2024), Pantelleria Volcano (Italy) (Picone et al. 2020), Golden Dome Cave (California, USA) (Maggiori et al. 2025), Mauna Loa Volcano (Hawaii, USA) (Gadson et al. 2022), and Svalbard (Norway) (Ricci et al. 2025). These sites span seven countries and encompass diverse environmental conditions, from hyperarid deserts and subglacial volcanoes to geothermal soils, providing a broad representation of volcanic habitats relevant to the search for extraterrestrial life (Fig. 1).

Publicly available MAGs from these sites were used as the source of genomic data (Table S1). A total of 212 MAGs were analysed to investigate the presence and diversity of CODH-related genes. To do this, custom databases were constructed prior to conducting BLASTx analysis. Reference CODH protein sequences were extracted from the GlobDB r226 (Speth et al. 2025) based on their KEGG annotations (Kanehisa et al. 2023, Kananen et al. 2025) and were used to build the three CODH-specific databases. The first included bacterial-type Ni-CODH sequences corresponding to K00198 (*cooS*) and K00196 (*cooF*). The second comprised archaeal-type Ni-CODH sequences annotated as K00192 (*cdhA*), K00195 (*cdhB*), K00193 (*cdhC*), K00194 (*cdhD*), and K00197 (*cdhE*). The third database represented MoCu-CODH and included sequences associated with K03518 (*coxS*), K03519 (*coxM*), and K03520 (*coxL*). Sequences from each functional annotation were dereplicated at 90% sequence identity with CD-HIT v4.8.1 default parameters (Li and Godzik 2006). In the case of the MoCu-CODH large subunits (CoxL), sequences were further split into form I (AYRCSFR) and form II (AYRGAGR), based on strict matching of their canonical motifs using SeqFu’s v1.22.3 (Telatin et al. 2021) grep command.

**Figure 2:**
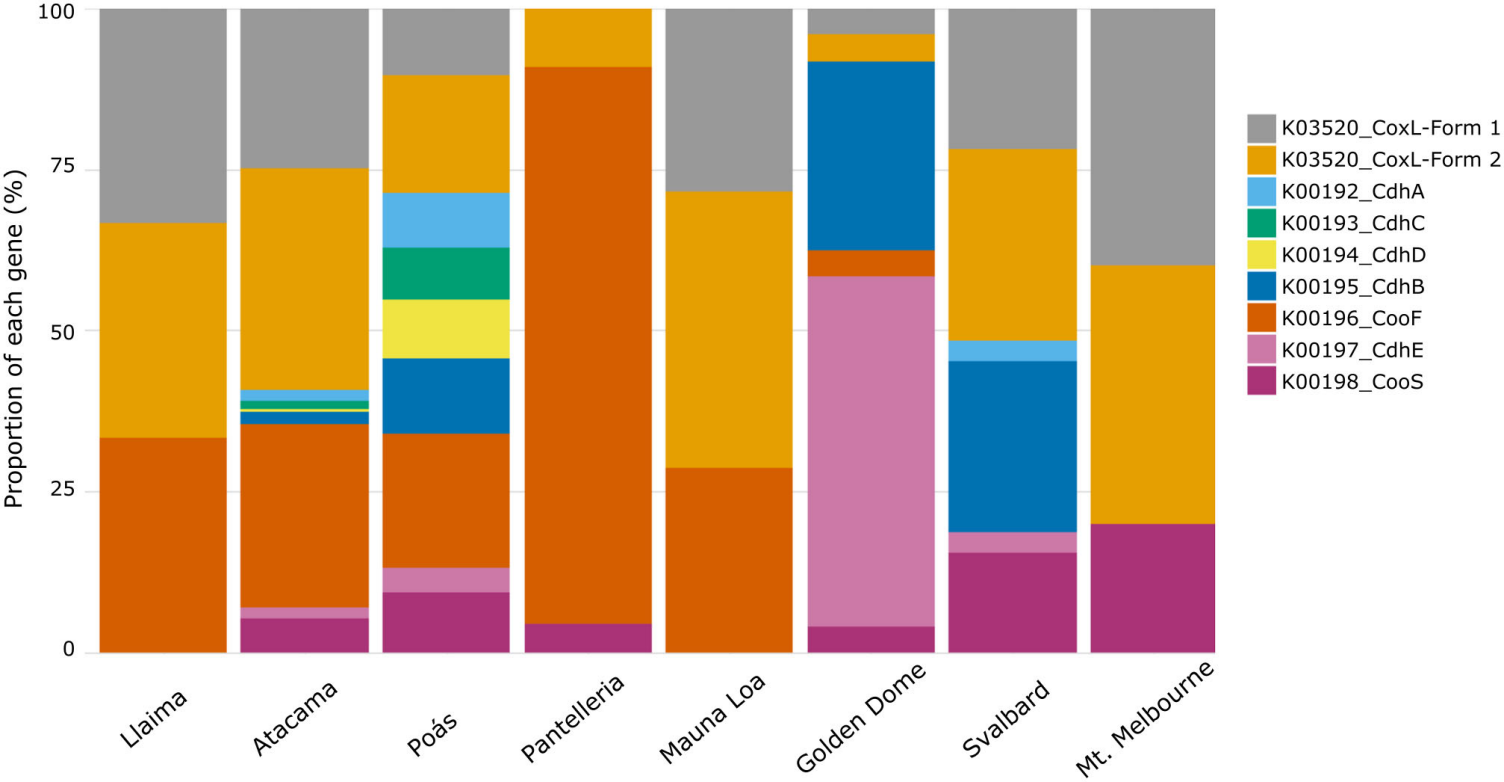
Relative abundance of Cdh/Coo and CoxL enzymes across eight global locations.

The analysis revealed distinct patterns in the presence, abundance, and sequence conservation of CODH (CoxL) and CODH/acetyl-CoA synthase (*cdh*/*coo*) gene clusters. The gene relative abundance profiles (Fig. 2) highlight clear differences in the dominance of these gene families, depending on the environmental context. In particular, the abundance of CoxL (K03520_CoxL-form I and K03520_CoxL-form II) was observed across most locations, specifically in the Atacama Desert, Mauna Loa, and Mt. Melbourne. These trends suggest that aerobic CO oxidation represents the primary energy acquisition pathway across these environments.

Other gene clusters tended to show location-specific prominence. The anaerobic *cooF* (K00196_CooF) was highly dominant in the Pantelleria Island sample. This result is associated with the dominance of the Bacillota phylum in this site and with the presence of thermoacidophilic and chemolithoautotrophic strains of *Hydrogenisulfobacillus filiaventi* and *Kyrpidia spormannii* (Table S2. MAGs: GCA_902809825.1_R50 and GCA_902829265.1_FAVT5). Similarly, the Golden Dome Cave soils exhibited a predominance of *cdhE* genes, indicating that anaerobic CO-dependent carbon fixation was a central pathway in these more carbon-poor or redox-variable environments. This *cdh* dominance in Golden Dome Cave aligns with a substantial presence of an unclassified species (Table S2. MAG: GCA_048569215.1_ASM4856921v1), potentially capable of reductive acetyl-CoA pathway utilisation. Crucially, gene abundance varied not only in terms of environmental context but also across phyla. For instance, sites like the Atacama Desert and Mauna Loa Volcano that showed high abundances of CoxL-related genes were also rich in Acidobacteriota and Actinomycetota phyla (Table S2). However, this trend was not observed at Mt. Melbourne. While the site was also rich in aerobic CO oxidation genes, the microbial community structure was predominantly composed of the Chloroflexota family Dormibacteraceae (MAG: SRR30404090) and the archaeon *Australarchaeum erebusense* (MAG: SRR30463440). Members of Dormibacteraceae have been shown to possess RuBisCO type IE and a functional CBB cycle, enabling atmospheric chemosynthesis and allowing them to fix CO₂ using energy derived primarily from CO oxidation in nutrient-poor environments (Montgomery et al. 2021). This supports the idea that, under different selective environments, microorganisms may express similar functions. Moreover, the observed correlation between the dominant CODH gene profile and the dominant microbial phylum at specific locations (e.g. Pantelleria’s CooF linked to Bacillota, and CoxL forms at Llaima, Atacama, Mauna Loa, and Mt. Melbourne linked to Acidobacteriota and Chloroflexota) suggests that similar functional capacities for CO metabolism are maintained across distinct microbial communities in different environmental contexts.

**Figure 3:**
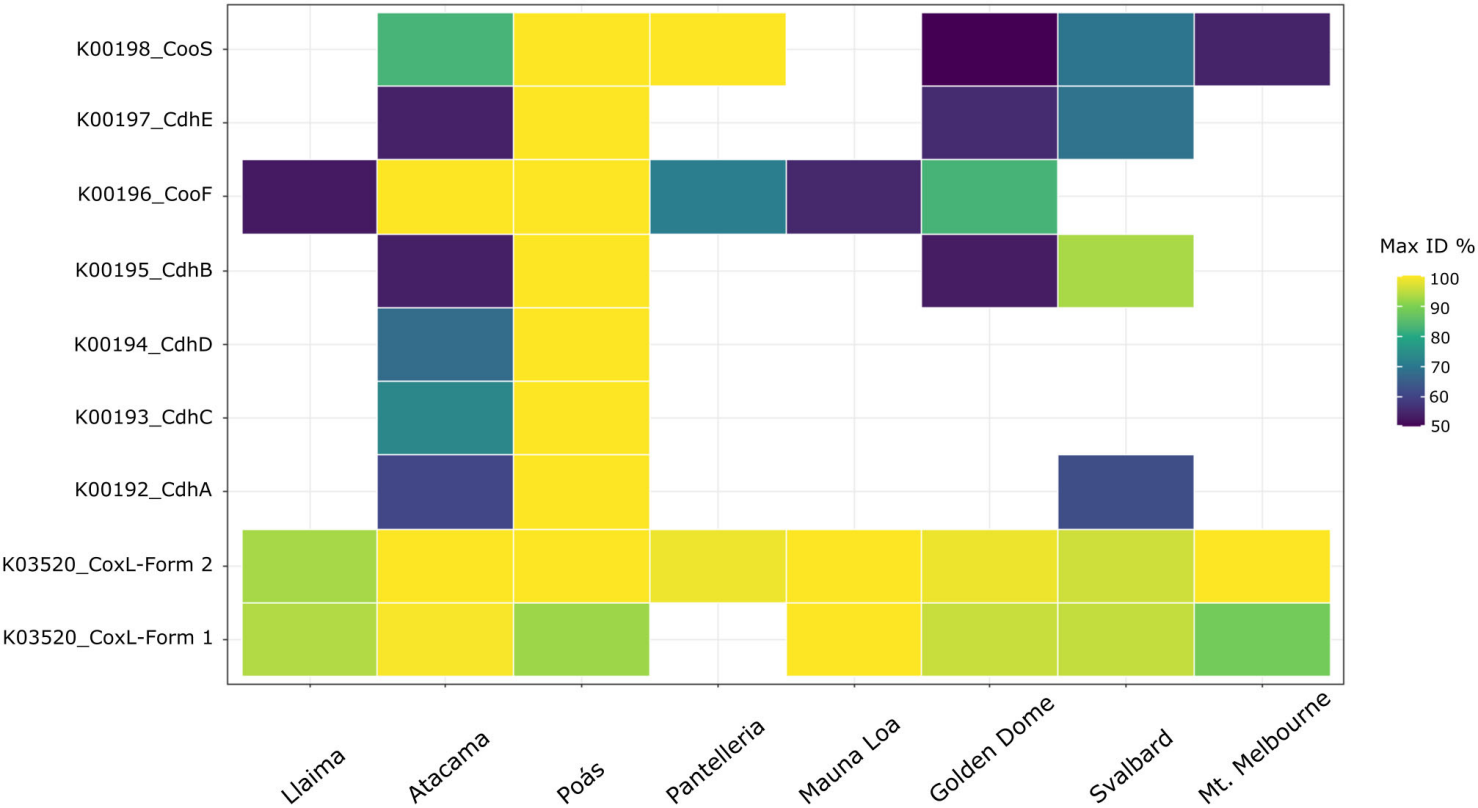
Maximum BLASTx identity (%) of key Cdh/Coo and CoxL enzymes across eight global locations. This heatmap illustrates the sequence conservation of each gene in different environments, ranging from highly conserved (bright yellow) to poorly conserved (dark blue).

Following BLASTx, a heatmap was generated to visualise the highest percentage identity (ID%) of hits to a reference gene database for each MAG (Fig. 3). These data further demonstrated contrasting evolutionary dynamics between gene families. For example, *coxL* sequences (K03520_CoxL-form I and K03520_CoxL-form II) were highly conserved across all environments, consistently reaching 90–100% identity, indicating strong selective pressure to maintain aerobic CO oxidation functionality. Here, the two forms were similarly distributed, with form II being more conserved than form I, except at the Pantelleria site, where only form II was detected.

Conversely, genes encoding for Cdh/Coo exhibited much greater variability in both presence and sequence identity, with high conservation limited to specific sites such as Atacama (K00196_CooF), Poás Volcano (from K00192_CdhA to K00198_CooS) and Pantelleria (K00198_CooS). *cdh* genes were less conserved in sites like Golden Dome Cave (K00196_CooF) and Svalbard (K00195_CdhB), suggesting localised adaptations of anaerobic CO fixation pathways. Such patterns likely reflect differences in substrate availability, oxygen exposure, and competing metabolic processes. The observed absence or low detection of some *cdh*/*coo* genes in Llaima, Pantelleria, Mauna Loa, and Mt. Melbourne highlights that these genes may not be universally required, and microbial communities may rely predominantly on CO oxidation in more oxygenated or thermally dynamic substrates.

These contrasting evolutionary and ecological patterns frame the interpretation of the Poás Volcano dataset, which represents a notable outlier in both gene abundance and metabolic versatility. Specifically, the higher gene abundances and conservation at Poás Volcano likely occurred due to the higher microbial diversity observed at this site, dominated by Acidobacteriota, Chloroflexota, and Pseudomonadota, along with minor contributions from other phyla. Notably, two species within the generally anaerobic sulfate-reducing phylum Desulfobacterota displayed striking gene versatility, with detectable hits for all nine CODH-related gene clusters associated with both anaerobic and aerobic metabolism (Table S2. MAGs: GCA_037440645.1_GCA_037440645.1_ASM3744064v1 and GCA_037440525.1_GCA_037440525.1_ASM3744052v1). Especially MAG GCA_037440525.1_GCA_037440525.1_ASM3744052v1 showed the highest functional gene abundances across all datasets, supporting the high potential of these microorganisms for CO oxidation.

## Conclusions

The study of CODH distribution in volcanic-origin soils that are considered astrobiological analogues highlights the integral role of trace gas metabolism in shaping early microbial ecosystems. Under diverse carbon-limited and geochemically dynamic conditions, distinct global patterns in the presence of CODH-encoding genes were observed. Aerobic MoCu-CODH, encoded by *coxL* genes, showed a broad distribution, indicating that aerobic CO oxidation may represent a metabolically stable strategy across a range of volcanic environments. In contrast, *cdh*- and *coo*-related genes were patchily distributed, possibly in a site-specific manner, highlighting both the evolutionary antiquity of CODH and its notable functional plasticity. We propose that these pioneering chemolithotrophs not only provide energy for survival but actively drive primary succession by converting inorganic CO into bioavailable carbon, thereby enabling the establishment of more complex microbial communities and laying the biochemical foundation for subsequent ecosystems. The exceptional gene versatility observed in taxa from Poás Volcano, spanning both anaerobic and aerobic CODH pathways, illustrates the potential for metabolic flexibility to support life under fluctuating environmental stresses. Our review demonstrates that volcanic soils host diverse CODH-encoding microorganisms whose metabolic strategies reflect the heterogeneity of volcanic habitats, providing insights into potential metabolic pathways that could support life on Mars and other extraterrestrial volcanic ecosystems.

## Supplementary Material

fiag022_Supplemental_Files

## Data Availability

Individual accession numbers for the metagenomic assemblies can be found in Supplementary Table S1.
