# Supplementary material for: Carbon monoxide dehydrogenase-encoding microorganisms in volcanic astrobiological analogues: an enzyme system to investigate the evolution of life
Source: FEMS Microbiol Ecol. 2026 Feb 25;102(4):fiag022. doi: 10.1093/femsec/fiag022 (PMC12996764; doi:10.1093/femsec/fiag022)
Supplement: fiag022_Supplemental_Files [file fiag022_supplemental_files.zip › Latorre_SI_final.pdf]

Supplementary information for

Carbon monoxide dehydrogenase-encoding microorganisms in volcanic astrobiological analogues: an enzyme system to investigate the evolution of life

Vito Latorre[1], Xabier Vázquez-Campos[2], Belinda Ferrari[2], and Marcela Hernández[1*]

[1] School of Biological Sciences, University of East Anglia, NR4 7TJ, UK

[2] School of Biotech and Biomolecular Science, University of New South Wales, Sydney, Australia

Corresponding author: Marcela Hernández, School of Biological Sciences, University of East Anglia, Norwich, NR4 7TJ, UK, marcela.hernandez@uea.ac.uk

Table S1

Table S1. Summary of the datasets included in this study, indicating sampling location, country of origin and corresponding Bioproject accession number.

**Location**	**Country**	**Bioproject**	**DOI**	**Reference**
Llaima	Chile	PRJNA602601	10.3390/microorganisms8121880	Hernández *et al*. 2020
Atacama	Chile	PRJNA291433	10.1128/msphere.00192-24	Andreani-Gerard *et al*. 2024
Poás Volcano	Costa Rica	PRJNA627197	10.1038/s41396-022-01331-7	Rogers *et al*. 2023
Mount Melbourne	Antarctica	PRJNA1126331	10.1038/s41597-024-03875-z	Myeong *et al*. 2024
Pantelleria	Italy	PRJEB36447	10.1128/msystems.00517-20	Picone *et al*. 2020
Golden Dome Cave	California	PRJNA1189542	10.1128/mra.01335-24	Maggiori *et al*. 2025
Mauna Loa	Hawaii	PRJNA818798	10.1128/mra.00556-22	Gadson *et al*. 2022
Svalbard	Norway	PRJNA1206715	10.1101/2025.02.19.639150	Ricci *et al*. 2025

References

Andreani-Gerard CM, Cambiazo V, González M. Biosynthetic gene clusters from uncultivated soil bacteria of the Atacama Desert. *MSphere.* 2024;**9**, e00192-00124. 10.1128/msphere.00192-24. https://doi.org/10.1128/msphere.00192-24.

Gadson O, et al. Metagenome-assembled genome of a putative chemoheterotroph from volcanic terrain in Hawaii. *Microbiol Resour Announc.* 2022;**11**: e00556-00522. 10.1128/mra.00556-22. https://doi.org/10.1128/mra.00556-22.

Hernández M, et al. Reconstructing genomes of carbon monoxide oxidisers in volcanic deposits including members of the class Ktedonobacteria. *Microorganisms.* 2020;**8**: 1880. https://doi.org/10.3390/microorganisms8121880.

Maggiori C. Draft genome sequence of a member of a putatively novel Rubrobacteraceae genus from lava tubes in Lava Beds National Monument. *Microbiol Resour Announc.* 2025;**14**: e01335-01324. https://doi.org/10.1128/mra.01335-24

Myeong NR, Choe Y-H, Shin SC, Kim J, et al. Genomic profiling of Antarctic geothermal microbiomes using long-read, Hi-C, and single-cell techniques. *Sci Data.* 2024;**11**: 1023. https://doi.org/10.1038/s41597-024-03875-z

Picone N, et al. Geothermal gases shape the microbial community of the volcanic soil of Pantelleria, Italy. *MSystems.* 2020;**5**: 10-1128. https://doi.org/10.1128/msystems.00517-20.

Ricci F, et al. Metabolically flexible microorganisms rapidly establish glacial foreland ecosystems. *bioRxiv* 2025;2025.02. https://doi.org/10.1101/2025.02.19.639150.

Rogers TJ, et al. Chemolithoautotroph distributions across the subsurface of a convergent margin. *ISME J.* 2023;**17**: 140-150. https://doi.org/10.1038/s41396-022-01331-7.
